# The Ying and Yang of Ganglioside Function in Cancer

**DOI:** 10.3390/cancers15225362

**Published:** 2023-11-10

**Authors:** Cara-Lynne Schengrund

**Affiliations:** Department of Biochemistry and Molecular Biology, The Pennsylvania State University College of Medicine, Hershey, PA 17033, USA; cxs8@psu.edu

**Keywords:** ganglioside, lipid rafts, growth factors, multivalent oligosaccharide ligands, glycocalyx

## Abstract

**Simple Summary:**

Gangliosides, sialylated glycosphingolipids, have been found to affect cell growth as a result of their effect on signaling pathways. An increasing number of studies have shown that cells isolated from different types of cancer may overexpress a specific ganglioside(s), which can affect the interaction of specific growth factors with their receptors, thereby altering cell behavior. As more is learned about the mechanisms by which gangliosides exert their positive or negative effects, it is anticipated that it will provide the basis for the development of treatments to inhibit the proliferation and/or metastasis of cancer cells.

**Abstract:**

The plethora of information about the expression of cancer cell-associated gangliosides, their role(s) in signal transduction, and their potential usefulness in the development of cancer treatments makes this an appropriate time to review these enigmatic glycosphingolipids. Evidence, reflecting the work of many, indicates that (1) expression of specific gangliosides, not generally found in high concentrations in most normal human cells, can be linked to certain types of cancer. (2) Gangliosides can affect the ability of cells to interact either directly or indirectly with growth factor receptors, thereby changing such things as a cell’s mobility, rate of proliferation, and metastatic ability. (3) Anti-ganglioside antibodies have been tested, with some success, as potential treatments for certain cancers. (4) Cancer-associated gangliosides shed into the circulation can (a) affect immune cell responsiveness either positively or negatively, (b) be considered as diagnostic markers, and (c) be used to look for recurrence. (5) Cancer registries enable investigators to evaluate data from sufficient numbers of patients to obtain information about potential therapies. Despite advances that have been made, a discussion of possible approaches to identifying additional treatment strategies to inhibit metastasis, responsible for the majority of deaths of cancer patients, as well as for treating therapy-resistant tumors, is included.

## 1. Introduction

The word “glycocalyx” introduced by Martinex-Palomo [1] provided researchers with a descriptive term to refer to the carbohydrate-rich coating of the cell’s plasma membrane. Initially identified by Luft [2], it is comprised of glycoproteins, proteoglycans, glycosaminoglycans, and glycolipids. The glycocalyx plays a role in such functions as the maintenance of cell shape, provision of binding sites for molecules, cells, and pathogens, cell recognition, and metastasis. In relation to cancer, the composition of the glycocalyx can affect the way in which cells interact with their environment. Because of the wide variety that can exist in the glycan composition of the glycocalyx, this review will focus on the potential role(s) in cancer of just one class of its components, gangliosides, sialic acid-containing glycosphingolipids having the ganglio-series of sugars (Galβ1-3GalNAcβ1-4Galβ1-4Glcβ1-), or portion thereof, linked to ceramide. Based on their carbohydrate components, over 200 gangliosides have been identified [3] since their initial discovery by Thudichum [4]. In addition to differences in their sugar composition, gangliosides can also differ in their lipid component, ceramide [5], which can also affect cell behavior [6]. The findings that the carbohydrate portion of gangliosides can affect cell growth and metastasis, e.g., [7], angiogenesis [8,9], and function as cancer cell antigens, e.g., [10] coupled with our nascent understanding of their role(s) in these processes make them the focus of this review centered on their effect on cancer cell behavior. It is important to fully understand at the molecular level changes responsible for the presence of gangliosides on cancer cells that are not found to any extent on mature, healthy cells, as well as how they may affect cell behavior.

Gangliosides can be found on the outer surface of plasma membranes [11], where they can be enriched in lipid rafts, discrete microdomains enriched in sphingolipids and cholesterol plus proteins that may be involved in signal transduction [12]. Structurally, gangliosides have been shown to contribute to membrane organization, with those having a more complex carbohydrate structure found in areas of the membrane with more curvature reflecting the relative volume occupied by their carbohydrate components that extend outward from the cell surface [13]. Changes in ganglioside composition can affect the association of lipid raft protein components such as receptor tyrosine kinases [14] as well as the ability of growth factors to interact with their cell surface receptors [15]. Studies have shown that, as a result, gangliosides can affect functions such as cell adhesion [16], mobility [17], differentiation [18,19], metastasis [20], and angiogenesis [21]. To answer the question of how gangliosides exert these effects research has increasingly focused on their role in signal transduction (PubMed lists ~950 publications on gangliosides and signal transduction between 1984 and May 2023). This review discusses (1) how alterations in ganglioside expression may disrupt signaling pathways, thereby causing changes in cell mobility, proliferation and/or survival and (2) how understanding the causes of altered ganglioside expression may serve as a prognosticator of tumor severity and provide a guide to possible treatment. 

## 2. Ganglioside Synthesis

**Figure 1:**
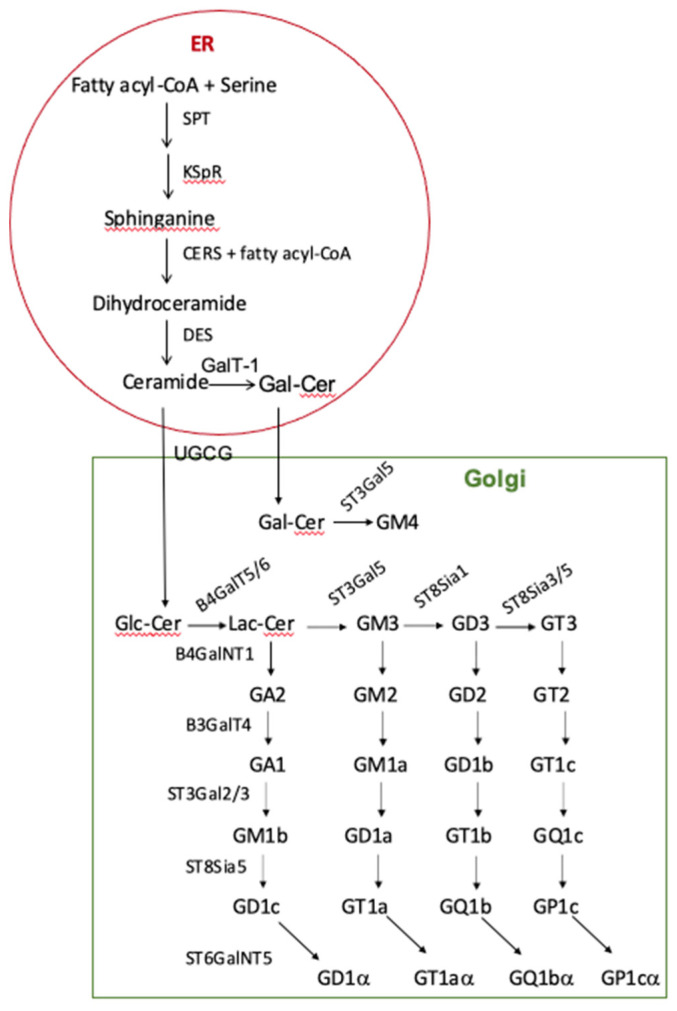
Outline of ganglioside synthesis. Ganglioside nomenclature used was initially developed by Svennerholm [29]. The steps involved in the endoplasmic reticulum (ER) are shown in the red circle, and those in the **Golgi** are in the green square. **ER**-associated enzymes are SPT, serine-palmitoyl transferase; KSpR, 3-ketodihydrosphingosine reductase; CERS, ceramide synthase; DES, dihydroceramide desaturase; and GalT-1, UDP-galactose:ceramide galactosyl-transferase. **Golgi**-associated enzymes are UGCG, UDP-glucose:ceramide β1-1′glucosyltransferase; ST3Gal5, ST3 β-galactoside α-2,3-sialyltransferase 5; B4GalT5/6, UDP-galactose:glucosyl-ceramide β1-4 galactosyl transferase (lactosylceramide synthase) [30]; B4GalNT1, UDP-GalNAc:LacCer/GM3/GD3/GT3 β1-4 N-acetylgalactoseaminyl transferase (ganglioside GA2, GM2, D2, and T2 synthase); B3GalT4, UDP-galactose: GA2/GM2/GD2/GT2 β1–3 galactosyl transferase (ganglioside GA1, GM1a, GD1b, and GT1c synthase); ST3Gal5, CMP-sialic acid:lactosyl-ceramide α2-3 sialyltransferase (GM3 synthase); ST8SIA1, CMP-sialic acid:GM3 α-2,8-sialyltransferase (GD3 synthase); ST8SIA3/5, CMP-sialic acid:GD3 α-2,8-sialyltransferase (GT3 synthase); ST8Sia5, similar specificity to ST8SIA3/5 [31]; and ST6GalNT5, ST6 N-acetylgalactosaminide α-2,6-sialyltransferase 5.

The simple schematic shown in Figure 1 provides a general outline for the synthesis of the ceramide portion of gangliosides as well as the carbohydrate portion (defined in Table 1) of a number of them. While the emphasis in this review is on the carbohydrate portion of gangliosides, the composition of the ceramide component may affect their ability to form nanodomains [22]. Ganglioside synthesis is not template-mediated but dependent on substrate availability, activity of transport proteins, glycosyltransferases, and modification of specific sugar residues. An example of a modified sugar is N-glycolylneuraminic acid, frequently found in cancer cells. While it can also be found in normal human cells, existing evidence indicates it is not synthesized by them, apparently due to a deficit in the key enzyme, CMP-N-acetylneuraminic acid hydroxylase [23,24,25]. For a review of ganglioside synthesis and the diversity of the oligosaccharide portion, see [26]. Once synthesized and trafficked to the plasma membrane, further modification can occur due to the activity of plasma-membrane-associated enzymes such as sialidase, Neu3 [27], and sialyltransferases such as ST8SIA1 [28]. Mutations affecting the functional expression of any of the proteins needed can result in abnormal ganglioside expression and altered behavior of the cells affected. When such errors occur, the altered ganglioside composition can exert positive or negative cellular effects by affecting things such as lipid raft composition, cell membrane structure, and signal transduction.

## 3. Gangliosides and Signal Transduction 

Since about the time when Bremer et al. [32] reported that GM3 inhibited both EGF and PDGF-stimulated mitogenesis while GM1 only affected that induced by PDGF, research into the effects of specific gangliosides on the response of transformed cells to the interaction of specific factors with their receptors has proliferated. Table 2 indicates gangliosides and some of the signal transduction pathways they have been reported to affect and possible cellular effect(s) induced. In some instances, the effect is initiated by a ganglioside affecting the interaction of a growth factor with its receptor. In others, the effect of the ganglioside may be mediated by its binding to another protein, which in turn alters the binding of the factor to its receptor [33,34]. It is anticipated that as more is learned about the mechanisms by which gangliosides affect signal transduction, it will provide the basis for the development of new drugs to inhibit the growth/metastasis of cancer cells. The basis for this possibility is the fact that many gangliosides found in relatively high concentration in cancer cells (e.g., GM3, GM2, GD3, and GD2) are not found in high concentrations in the brain, which predominantly expresses GM1, GD1a, GD1b, and GT1b [35,36]. In addition, access to circulating compounds in the brain is limited by the blood–brain barrier [37], so inhibiting an interaction that occurs due to the over-expression of a specific ganglioside by cancer cells is less likely to affect it. The importance of cancer-associated gangliosides as possible therapeutic targets is supported by findings from a National Cancer Institute pilot project for the acceleration of translational research. That report ranked GD2 12th out of 75 possible cancer cell-associated compounds recommended for study. Also included, albeit lower in the list, were fucosylated GM1 (fucose linked α1-2 to the terminal Gal of GM1a, associated with small cell lung cancer [38]), GM1, and GD3 [39]. For a recent review of the roles of GD2 and GD3 in cancer, see Cao and colleagues [40].

In many instances, there are multiple members in the growth factor’s family, and when defined in the reference cited, it is listed in Table 2 under pathway affected. Failure to specify may reflect the fact that specific members were identified subsequent to when the cited research was conducted (e.g., identification of 22 *FGF* genes [63] and multiple isoforms of VGEF-A [64]). As more is learned about the families of specific growth factors, it may become necessary to categorize the effects of each as they may be different [65]. This is underscored by the observation that while both FGF1 and FGF2 reduced skin flap necrosis and ischemia in a rat model, FGF2 was more potent [66]. While the effect(s) of specific gangliosides on signal transduction induced by specific growth factors are being identified, analyses have indicated that not all cancer cells respond in the same manner to drugs used to disrupt those interactions. This may reflect somatic mutations such as those identified for the EGF receptor in glioma cells [67,68]. Interestingly, studies of non-small cell lung cancer have shown that east Asian populations have a higher prevalence of somatic mutations in the EGF receptor than Caucasians [69,70], an observation that led researchers to suggest that the reason Asians may be more sensitive to tyrosine kinase inhibitors is due to mutations that reduce expression of the EGF receptor [71]. 

## 4. Altered Expression of Proteins Required for Ganglioside Synthesis in Cancer Cells

Alterations in the expression of gangliosides in cancer cells have led to the identification of changes in the expression of proteins necessary for their de novo synthesis from GlcCer. In some instances, the specific mutations/epigenetic modifications responsible have been identified, as well as whether they correlate with patient prognosis and survival (see Table 3). In addition to the Golgi-associated synthetic enzymes shown, glycosidases such as sialidase (Neu3 [72]), an ecto-enzyme present on the plasma membrane of both normal and transformed cells [73], can affect ganglioside composition as it preferentially catalyzes cleavage of sialyl residues from gangliosides with the exception, in the case of human sialidase, of GM1 and GM2 [27]. Its high specificity for ganglioside substrates is in contrast to that of sialidases NEU1, 2, and 4 [74]. Interestingly, increased levels of NEU3 mRNA have been found in prostate [75], colon, renal and ovarian cancers [72] and to be down-regulated in acute lymphoblastic leukemia [76] and glioblastoma [77] correlating with enhanced invasion and migration. Evidence indicates that NEU3 may activate the EGF receptor by catalyzing the removal of sialic acid residues [78]. In contrast to glycosidases, plasma-membrane-associated glycosyl transferases can catalyze the addition of sugars from their nucleotide donor to cell surface gangliosides [79]. For example, ST8Sia1 (ecto-ST8Sia1) can catalyze the sialylation of GM3 to yield GD3 [80], a tumor-associated carbohydrate antigen [10]. 

In addition to observations indicating that the expression/activity of a protein can vary depending on cancer type, resultant cell behavior can also reflect the model system studied. For example, BrM2 cells, cells derived from a triple-negative breast cancer cell line that overexpressed ST6GALNT5, could transmigrate through an in vitro barrier model comprised of HUVECs [81] while the same cells showed poorer adhesion and no change in transmigration compared with controls when an in vitro human blood–brain barrier (BBB) model was used [82].

**Table 3:** Examples of altered enzymatic activity and resultant ganglioside expression in cancer cells and possible correlation with prognosis.

Enzyme ^a^	Problem	Ganglioside ^b^	Correlation with Cancer Prognosis ^c^	Reference ^d^
UGCG	↑ Expression	Glc-Cer	+ metastasis and chemotherapy resistance	[83]
B4GALT5 ^e^	↑ mRNA & expression	Lac-Cer	+ survival	[84]
B4GALNT1	↑ Expression	↑ GM2	Depends on tumor type	[85]
B3GALT4	↓ Activity	↑ GD2	+ survival from neuroblastoma	[86]
ST3GAL2	↑ mRNA	↑ GD1a?	+ progression	[87,88,89]
ST3GAL5	↑ Expression	↑ GM3	+ poor prognosis for ccRCC ^f^	[90]
ST8SIA1	↑ Expression	↑ GD3	+ poor outcome neuro-ectodermal cancers + tumor growth and metastasis in breast cancer	[28]
				[91,92,93]
ST8SIA5	↓ Expression	↓ GT3, GD1c,GT1a GQ1b	+ poor survival colon cancer	[94]
ST6GALNT5	↑ Expression	↑ GD1α	+ decreased adhesion of human BrM2 cells to an in vitro BBB model	[82]
NEU 3	↑ Expression	↑ GM1, GM2, GM3	+ renal cell carcinoma	[95]

^a^ Enzyme abbreviations are defined in the legend to Figure 1. ^b^ Ganglioside listed is a product of the reaction catalyzed. ^c^ + indicates a positive correlation. ^d^ Sample references are given. ^e^ B4GalT5/6 was found to be expressed in genes needed for myelin formation in mice [30], while in another study, only B4GalT5 was needed [96]. ^f^ Abbreviations: ccRcc, clear cell Renal Cell Carcinoma; BrM2, MDA-MB231 breast cancer cells.

While this review has focused on the carbohydrate portion of gangliosides and the aberrant expression of specific glycosyl transferases, it is necessary to discuss the ceramide component, which may affect ganglioside packing in the membrane, which in turn may alter function [22,97]. A key enzyme in ceramide synthesis is CerS, which has been found to consist of six different entities with differing but overlapping fatty acid specificities [98] and whose activity may be regulated by their degree of phosphorylation [99]. For a review describing the enzymatic synthesis of ceramide and its effect on cancer, see [100]. While the ceramide composition of gangliosides isolated from brains of the young is predominantly d18:1/18 with d20:1 increasing with age [5], analysis of the ganglioside composition of glioblastoma multiforme (GBM) led to identification of high proportions of GD3, GT1, and GT1c with measurable amounts of the ceramide moiety (d18:1/24:1). Based on these results, GT1c containing ceramide (d18:1/24:1) was postulated to be a marker for GBM [101]. Once synthesized, ceramide serves as the precursor for the synthesis of GlcCer, which in healthy cells tends to have nonhydroxylated shorter-chain fatty acids and GalCer enriched in very long-chain α-hydroxylated fatty acids [102]. The composition/length of the fatty acid component of ceramide per se has been postulated to affect the membrane and, as a result, specific biological functions [103]. The variability in the ceramide component of gangliosides may reflect the degree of substrate specificity of enzymes required for their synthesis [26].

## 5. Effects of Circulating Gangliosides Shed from Tumor Cells

It is well known that tumor-associated gangliosides can be shed into the circulation, where they have been shown to correlate with relapse, incidence, rate of tumor progression [104], and immune responsiveness [105]. Circulating shed gangliosides can be used as diagnostic markers, for example, GM3 in the serum of patients with breast cancer [106] and GD2 for recurrence of neuroblastoma [107]. 

In terms of the effects of shed gangliosides on immune responsiveness, a 2023 review [55] includes a table (2) listing gangliosides expressed and shed by different tumor types and another describing the effect of specific gangliosides on the tumor as well as the immune response. To summarize reports cited in that table about the effects of gangliosides on immune cell responses: “shed” GM3, GM2, GM1, GD3, and GD1a had pro-tumor effects on monocytes, macrophage, T cells, and dendritic cells on which GD2 also had a pro-tumor effect. In addition, “shed” GD2, GD1b, and GT1b were reported to have both anti- and pro-tumor effects on T cells, depending on tumor type. Results reported for the effects of gangliosides on B and NK/NKT cells indicated that “shed” GM2, GD1b, and GT1b induced a pro-tumor effect and GD1a had an anti-tumor effect on B cells while exposure to “shed” GM2 and GM1 induced pro-tumor responses in NK/NKT cells and GD3 induced reactions that in some instances were pro-tumor and others, anti-tumor. “Shed”GM3 (GM3 added in medium) was reported to be pro-tumor, while when tumor cell-associated, it induced an anti-tumor response by the NK/NKT cells. Most of the literature cited by van der Haar Avila et al. [55] reported experiments performed using added purified gangliosides (hence the use of quotations around shed) to “mimic” the effects of those that would actually be shed into the circulation by tumor cells. This might be problematic because experiments have shown that gangliosides shed into the circulation by tumor cells can be found in membrane vesicles, micelles, and as monomers [108]. This raises the possibility that gangliosides present in membrane vesicles may induce different responses than those fed to cells in serum, which, depending upon concentration, might be present as monomers [109]. In contrast to monomeric gangliosides, those in micelles would present “multivalent” ligands to target cells, as might those in membrane vesicles.

A number of sialic acid-binding immunoglobulin-like lectins, siglecs, found primarily on cells of the immune system, can bind tumor cell-associated gangliosides. This can result in immunosuppression, permitting continued tumor growth [110], and other studies have shown it to enhance or inhibit the effect of gangliosides on immune cell responsiveness [111]. As of 2021, researchers had identified 15 human siglecs and the diseases with which they are associated [112]. Based on sequence similarity, siglecs have been divided into two subgroups: sialoadhesions (siglecs 1, 2, 4, and 15) that are distantly related [110] with the rest that are more closely related [110] in the CD33 group (siglecs 3, 5, 6, 7, 8, 9, 10, 11, and 14). Gangliosides identified as ligands for siglecs 1-5 and 7-10 are shown in Table 4. Because binding of a siglec to a ganglioside tends to be low-affinity [113], like many protein–carbohydrate interactions [114], clustering of both siglec and ganglioside is needed in order to have high affinity binding [115]. Because the presence of gangliosides in membrane vesicles and in micelles could provide multivalent ligands for binding by siglec clusters, care should be taken when using added gangliosides in experiments designed to address the question of siglec binding to shed tumor-associated ones. While the functions of siglecs 4 and 7 are relatively well-defined, there is still research that needs to be pursued to understand those of the others. As more has been learned about siglec–ganglioside interactions, investigators have used knowledge of selectin binding for potential therapeutic purposes such as active targeting of siglec 1 (CD169)-expressing antigen-presenting cells (APCs) and enhancement of chimeric antigen receptor T (CAR-T)-cell activation using GM3-containing nanoparticles [116], and ganglioside-containing liposomes to boost patients’ anti-tumor CD8^+^ T cell responses [117].

## 6. Use of a Cancer-Associated Ganglioside in the Development of an Anti-Ganglioside Antibody Cancer Therapy

While monoclonal antibodies were produced in the 1970s [126], it took decades before clinical trials indicated that they might be useful in the fight against cancer. That the use of monoclonal antibodies, in general, was still questionable almost 40 years later can be seen in a 2013 article in *Science* justifying the possible use of cancer immunotherapy as the breakthrough of the year [127]. The finding of specific cancer-associated gangliosides, such as GD2 in neuroblastoma and melanoma cells, but not to any extent in most mature untransformed cells [128,129] supported the idea of applying the use of anti-ganglioside antibodies as a possible cancer treatment [130]. A problem encountered with the use of intact anti-GD2 antibodies for the treatment of neuroblastoma is that when bound by receptors recognizing its fragment crystallizable portion (Fc or tail region), it often causes a relatively opioid-resistant non-neurotropic pain (allodynia) that tends to clear when the treatment is stopped [131]. The findings of the clinical studies led to approval by the FDA in 2015 for the use of Dinutuximab, a human/mouse chimeric monoclonal anti-GD2 lgG (ch14.18) antibody, in the treatment of patients with high-risk neuroblastoma while the European Medicines Agency approved the use of Dinutuximab beta (ch14.18/CHO). Analysis of the data obtained from the study of over 1000 stage 4 neuroblastoma patients treated with Dinutuximab indicated that it did improve 5-year event-free survival as well as overall survival [132].

To try to reduce side effects seen in patients treated with Dinutuximab, pegylated monomers of the fragment antigen-binding (Fab) region of the antibody were synthesized and tested. They caused less pain, and their binding was comparable to that seen with the single-chain variable fragment (scFv). To determine whether multivalency would enhance binding, as found in studies of the binding of cholera and Shiga toxin to carriers of different numbers of the oligosaccharide portion of GM1 [133,134], investigators looked at the effect of using dimers and tetramers of the scFvs. A significant increase in affinity for GD2 was seen using either multimer [135]. Multivalency also resulted in an increase in circulation time, increased penetration into tumors in mice, and enhanced cytotoxic effects on GD2-expressing tumor cells [136]. 

While the use of a tumor antigen-directed antibody appears to be a direct approach, additional research indicates that there may be confounding variables. For example, in a study of 53 neuroblastoma patients given a long-term infusion of Dinutuximab, it was observed that those having high-affinity Fc gamma receptors 2A and 3A genotypes had a higher level of antibody-dependent cell-mediated cytotoxicity and better event-free survival than those having a low Fc gamma receptor genotype. Similarly, better results were obtained for patients having the killer cell immunoglobulin-like receptor/ligand haplotype B, compared with the inhibitory haplotype A and patients with both did best [137].

Because GD2 is expressed in a number of different cancers (e.g., neuroblastomas and melanomas [130], small cell lung cancer [138], and Ewing’s sarcoma [139]), investigators are testing additional approaches for using it therapeutically. An example of this is the use of GD2-specifc chimeric antigen receptor T cells for the treatment of small cell and non-small cell lung cancer [140]. The low expression of GD2 by normal tissues makes it a viable target for this approach.

In a somewhat different approach, the oligosaccharide portions of GD2 or GD3 were linked to PAMAM dendrimers having either two or four arms and used to vaccinate mice. They were then challenged with EL4-GD2+ lymphoma cells, and the tumors were found to grow more slowly than those in challenged unvaccinated animals [141].

## 7. Additional Questions That Need to Be Further Interrogated Relative to Potential Treatment of Patients with a Ganglioside-Characterized Cancer

Analysis of information about deaths of cancer patients indicated that the majority occurred as the result of cancer metastasis [142]. In addition to trying to improve upon immunotherapeutic approaches, mediating growth factor responses and changes in ganglioside synthesis, identification of additional treatment strategies to inhibit metastasis as well as for treating therapy-resistant tumors are needed. Examples of additional approaches either under or up for consideration when treating ganglioside-characterized cancer include the following: (1) interrogating controls on the expression of proteins that could cause aberrant glycosylation, more specifically sialylation, a characteristic seen in a number of cancers, and whether they might be therapeutic targets [143]; (2) identification of immune cell-specific checkpoints that might be effectively inhibited [144]; (3) development of effective inhibitors of angiogenesis vital for tumor growth; (4) use of databases to identify characteristics of cancers that respond favorably to treatment; and (5) characterization of the effect of oligosaccharide multivalency on immune responsive cells and how it might be used in the delivery of chemotherapeutic agents. The examples discussed are meant to allow readers to think about questions that need to be interrogated about the ganglioside-characterized cancer they are studying. 

### 7.1. Expression of Proteins That Might Cause Aberrant Glycosylation

An example of why it is necessary to understand controls on the synthesis as well as activity of the protein of interest is provided by studies of treatment-resistant prostate cancer cells that express GD2. Results indicated that the nuclear transcription factor kappa B (NFκB), a key subunit of which in its alternate pathway is RelB, enhanced expression of ST3Gal l, ll and synthesis of GD1a as well as ST3Gal Vl and synthesis of sialoparagloboside [145]. More specifically, identification of the mammalian NFκB homolog that significantly inhibited expression of all three sialyltransferases as well as their products, GD1a and sialoparagloboside, was confirmed when their synthesis by cancer cells exposed to siRelB was significantly reduced [145]. That the alternative RelB pathway is active in a number of different cancers can be seen in the following research findings: In a different study of prostate cancer, downregulation of RelB with siRelB reduced tumor growth [146], as did silencing of RelB in colorectal adenocarcinoma cells [147]. Results of studies of estrogen receptor-negative breast cancer cells indicated that expression of GD3 synthase in breast cancer cells was upregulated by the tumor necrosis factor via the NFκB pathway and, when overexpressed, was accompanied by increased tumor growth [148]. A large subset of RelB-positive patients with diffuse large B-cell lymphoma were found to have a poor response to immuno- and chemotherapy and enhanced expression of the cellular inhibitor of apoptosis protein 2 [149]. The later observation caused the authors to suggest that RelB activation might be used as a prognostic indicator. 

### 7.2. Gangliosides and Immune Checkpoint Inhibitors

According to the National Cancer Institute [https://www.cancer.gov/about-cancer/treatment/types/immunotherapy/checkpoint-inhibitors, accessed on 29 October 2023], an immune checkpoint inhibitor (ICI) functions by blocking binding by proteins, such as siglecs on the surface of T cells, to their ligands expressed on the cancer cells. This, in turn, sends an “off” signal to the T cells, just as it would if it bound to sialic acid-containing ligands on normal cells, and blocks the system from killing the cancer cells. The seven checkpoint inhibitors approved for use by the Food and Drug Administration were not developed against siglecs but against cytotoxic T lymphocyte antigen 4, programmed cell death protein 1, and programmed cell death protein 1 ligand 1 [150]. While clinical success has been seen, it was reported that across all cancers, only about 25% of patients responded positively to ICIs [151]. While no siglec ICIs have been approved for clinical use, the expression of sialylated cancer cell siglec ligands combined with the restricted siglec distribution on immune cells (Table 4), plus their effect on host–pathogen interactions, has supported the idea that siglecs are potential therapeutic targets for the development of ICIs [121]. An example of research in this area is provided by studies of two siglecs, 7 and 9, that recognize gangliosides (Table 4) in addition to other sialic-acid-containing ligands [152]. The studies were conducted using a humanized murine model generated on a siglec E KO background (siglec E is the murine counterpart to siglec 9 in humans). The results indicated that treatment with Fc-engineered anti-siglec 7 and anti-siglec 9 blocking antibodies reduced tumor burden [153]. 

In another study performed, tumors were treated with anti-CD47 and anti-GD2 antibodies using syngeneic and xenograft mouse models of GD2-characterized cancers (neuroblastoma, osteosarcoma, and small cell lung cell cancer). Anti-GD2 was used because of its use to treat neuroblastoma patients, and anti-CD47 because it can bind CD47, a checkpoint inhibitor expressed on cancer cells. When CD47 binds to its receptor, the signal regulatory protein alpha (SIRPα) on macrophages inhibits their activity. By using antibodies to both, binding of siglec 7 to GD2 was blocked, and there was an upregulation of cancer cell surface calreticulin, a pro-phagocytic signal, while by blocking binding by CD47 to SIRPα, macrophage remained active [154]. In the mouse neuroblastoma model, the combination eradicated tumors, while the same treatment of osteosarcoma and small cell lung cancer reduced tumor burden and lengthened survival times [154]. The results of these studies support considering a multipronged approach when considering the use of immune checkpoint inhibition as a potential treatment.

### 7.3. Gangliosides and Angiogenesis

Angiogenesis was first postulated by Folkman [155] to be essential for tumor development and that inhibiting it could inhibit a tumor’s growth. Subsequently, it was found that angiogenesis induced in the cornea by either prostaglandin E1 or basic fibroblast growth factor could be reduced by the addition of GM3 and enhanced by added GD3 [156]. Observations that gangliosides could affect angiogenesis led to a number of studies, some of which were performed using animal models of malignant brain tumors [for a review, see [157]. Subsequently, results of analyses of the ganglioside composition of tissues from two different types of human brain tumors, glioblastoma multiforme (GBM) [101] and anaplastic ganglioglioma [158], showed that both had high proportions of GD3. More specifically, GD3 concentration was more than 50% in anaplastic gangliogliomas, while in peritumoral and healthy brain tissue, it was <10% [158]. In a sample from a human patient with GBM, GD3 and GT1 each accounted for 36% of the ganglioside content [101]. The ceramide composition of gangliosides from both types of tumors exhibited heterogeneity, with the fatty acid C24:1 found in GD3 and GT1 in GBM and C24:0 most abundant in GD3 from anaplastic ganglioglioma tissue [158]. Interestingly, using performance ion mobility separation mass spectrometry, Sarbu et al. [101] identified 160 distinct components with high variability found in both the carbohydrate and ceramide components, adding another possible degree of complexity to the identification of ganglioside function. In unrelated studies, the length of the fatty acid chain in the ceramide component was shown to affect ganglioside binding by anti-ganglioside antibodies [159] as well as immunosuppressiveness [160]. The finding of elevated levels of GD3 in these human brain tumors supports the hypothesis that GD3 and GD3 synthase might be good therapeutic targets for the treatment of brain tumors refractory to other approaches, e.g., [161]. 

### 7.4. Potential Uses of Cancer Data Bank Information to Guide Research/Treatment

The advent of information technology has permitted the formation of databases that can be used to identify genetic differences between normal and cancer cells, as well as predictions of possible drug targets and treatment effectiveness. Examples of two sets of databases of freely available genomic information for a number of different cancers are The Cancer Genome Atlas (CGA) [https://www.cancer.gov/ccg/] and The Therapeutically Applicable Research to Generate Effective Treatments (TARGET) [https://www.cancer.gov/ccg/research/genome-sequencing/target]. The CGA provides genomic information for 33 cancer types, while TARGET provides it for seven different childhood cancers. Both were developed by the National Cancer Institute and are available for researchers to use. In addition to data banks containing genomic data, information about treatment and outcomes obtained from hospital registry data submitted by the Commission on Cancer-accredited facilities is also available [https://www.facs.org/quality-programs/cancer-programs/national-cancer-database]. This information can provide guidance when deciding upon treatment. A recent review provides an analysis of databases that are freely available, data that can be obtained, limitations of available databases, additional information needed, and how to use data to predict challenges that can be encountered during clinical development [162]. An example of the usefulness of these types of databanks can be seen in the evolution of treatment for patients with neuroblastoma that is refractory to surgery and more traditional forms of chemotherapy [163]. Information about drug efficacy in the treatment of different cancers with errors affecting the same protein can be helpful in determining when a new or improved drug may be more effective, e.g., [164]. 

### 7.5. Use and Possible Advantages of Multivalency When Targeting Binding to Saccharides

Multivalency is being applied in research looking at methods for delivering drugs to tumor cells that express unique carbohydrates on their cell surface. The presence of tumor-associated molecules, such as gangliosides GD2 and GD3 not found to any extent on normal tissue, provides the basis for investigating the use of carriers derivatized with appropriate ganglioside antibodies to deliver drugs to them. Identification of the benefits of using multimers of scFvs instead of intact anti-GD2 antibodies [135,136] supports the use of smaller targeting proteins that bind well and may be more effectively taken up by cells. Points to consider when investigating this approach for drug delivery to cancer cells include the following: the specificity of the tumor target, whether the target is found on healthy cells, and is it the only or predominant moiety recognized by the binding protein; is it affected by the number of binding antibodies/number of receptors expressed on the surface of target cells; is the carrier taken up readily by tumor cells and the drug readily released within them; how long does the drug remain effective; are other cells affected by released drug; do patients that fall into different classifications of the cancer respond similarly; and how easily can the particles be made, purified, and transported. As of 2023, 14 antibody-drug combinations had received FDA approval, and many more protein–drug combinations are under investigation [165]. An example of this approach is the use of a humanized murine IgG4 anti-CD33 (siglec 3) antibody to target gemtuzumab ozogamicin (GO) in acute myeloid leukemia cells. Seven years after approval, anti-CD33-GO was withdrawn from use because it might induce liver toxicity and veno-occlusive problems, as well as a lack of confirmation about clinical benefits during induction and maintenance. The seven years after its removal saw the accumulation of new data that resulted in its reapproval for use in treating a more defined set of patients with acute myeloid leukemia (for a review, see [166]). 

Recently, results were published describing the use of anti-GD2 (ch14.18) carrying a microtubule depolymerizing agent (monomethyl auristatin E or F) for the treatment of EL-4 lymphoma and B78-D14 melanoma in syngeneic mouse models. Significant inhibition of tumor growth was seen in both [167]. The finding that anti-GD2-monomethyl auristatin E or F was effective on two different types of GD2-expressing tumors supports interrogating this approach on other ganglioside-expressing cancers. 

## 8. Summary

While much has been learned about the biological roles of gangliosides in cancer, it is clear from this review that many questions still need to be answered. This is especially true considering that cancer incidence projections for the United States indicate that despite the fact that cancer rates have stabilized and deaths due to cancer have decreased due to aging of the population, the annual number of cancer cases is projected to increase by 49% to over 2 million annual cases by 2050 with most of them occurring in the elderly (≥75 years old [142]). 

Despite the success that chemotherapy and immunotherapy have seen with regard to treating cancer, it is evident that a subset of individuals with a specific type of cancer may prove refractory to chemotherapy, radiation, and/or immunotherapy treatments. Even when a “new” treatment such as Dinutuximab is introduced, questions about how to use it most effectively may still arise, e.g., [168]. This leads to the obvious question of how to use all of the information available to maximize the probability of obtaining effective treatments from the growing list of predictive and prognostic factors. Based on the information published and organized in databases, artificial intelligence (AI) is considered a potential tool for using this information to improve patient care. To computationally meet the complexity of the biological system and provide optimum individual care, AI has to incorporate all available data for both the patient and their cancer (for a recent review, see [169]). Because information about specific cancers is needed for AI to be developed, more data concerning items such as cell morphology, genetic and epigenetic information, treatment protocols, patient reactions to drugs, metabolic changes, protein structures, etc., is essential. In the interim, the additional information provided by basic research and clinical trials will continue to be incorporated into appropriate databases and ongoing treatment protocols.

## Figures and Tables

**Table 1:** Saccharide composition of gangliosides shown in Figure 1.

Ganglioside	Saccharide Composition
GA2	GalNAcβ1-4Galβ1-4Glcβ1- ^a^
GA1	Galβ1-3GalNAcβ1-4Galβ1-4Glcβ1-
GM1b	Neu5Acα2-3Galβ1-3GalNAcβ1-4Galβ1-4Glcβ1- ^b^
GD1aα	Neu5Acα2-3Galβ1-3(Neu5Acα2-6)GalNAcβ1-4Galβ1-4Glcβ1-
GM3	Neu5Acα2-3Galβ1-4Glcβ1-
GM2	GalNAcβ1-4(Neu5Acα2-3)Galβ1-4Glcβ1-
GM1a	Galβ1-3GalNAcβ1-4(Neu5Acα2-3)Galβ1-4Glcβ1-
GD1a	Neu5Acα2-3Galβ1-3GalNAcβ1-4(Neu5Acα2-3)Galβ1-4Glcβ1-
GT1a	Neu5Acα2-8Neu5Acα2-3Galβ1-3GalNAcβ1-4(Neu5Acα2-3)Galβ1-4Glcβ1-
GD3	Neu5Acα2-8Neu5Acα2-3Galβ1-4Glcβ1-
GD2	GalNAcβ1-4 (Neu5Acα2-8Neu5Acα2-3)Galβ1-4Glcβ1-
GD1b	Galβ1-3GalNAcβ1-4 (Neu5Acα2-8Neu5Acα2-3)Galβ1-4Glcβ1-
GT1b	Neu5Acα2-3Galβ1-3GalNAcβ1-4 (Neu5Acα2-8Neu5Acα2-3)Galβ1-4Glcβ1-
GQ1b	Neu5Acα2-8Neu5Acα2-3Galβ1-3GalNAcβ1-4 (Neu5Acα2-8Neu5Acα2-3)Galβ1-4Glcβ1-
GT3	Neu5Acα2-8Neu5Acα2-8Neu5Acα2-3Galβ1-4Glcβ1-
GT2	GalNAcβ1-4(Neu5Acα2-8Neu5Acα2-8Neu5Acα2-3)Galβ1-4Glcβ1-
GT1c	Galβ1-3GalNAcβ1-4(Neu5Acα2-8Neu5Acα2-8Neu5Acα2-3)Galβ1-4Glcβ1-
GQ1c	Neu5Acα2-3Galβ1-3GalNAcβ1-4(Neu5Acα2-8Neu5Acα2-8Neu5Acα2-3)Galβ1-4Glcβ1- ^c^

^a^ Each saccharide is linked to ceramide. ^b^ NeuAc refers to N-acetylneuraminic acid, the form of predominant sialic acid found in people. ^c^ GD1aα, the only α ganglioside for which the saccharide composition is listed, has a Neu5Ac residue linked α2–6 to GalNAc, a linkage found in each of the α gangliosides.

**Table 2:** Examples of growth factor signaling pathways affected by specific gangliosides.

Pathway Affected	Ganglioside	Effect	Cell Type	Reference
↑ ^a^ Akt ^b^ activity	GlcCer	↑ Proliferation	Breast cancer	[41]
↓ Proangiogenic effects of VEGF/VEGFR -2 and GD1a	GM3	↓ Angiogenesis	HUVECs ^c^	[8,9]
↓ EGFR phosphorylation	GM3	↓ Mitogenesis	Swiss 3T3 Human epidermoid carcinoma	[18,32,34]
FGF	GM3	↓ Proliferation	BHK and Swiss 3T3	[32,42]
FGF2	GM3	↑ Proliferation	Bovine aortic endothelial	[43]
↓ Dimerization of PDGFR	GM3	↑ Proliferation	Human glioma	[44]
uPA ↑ P70S6 kinase signaling	Over-expressed GM3	↑ Proliferation	Carcinoma SCC12	[45]
↑ EGFR kinase	de-N-Acetyl-GM3	↑ Proliferation	Melanoma	[46]
Binds integrin receptor ↑ FAK, Erk and Src phosphorylation	GM2	↑ Migration	Renal carcinoma	[33]
TGF-b1	GM2	↑ Growth and invasiveness	Pancreatic ductal adenocarcinoma	[47]
GM1 binds TrkA	GM1	↑ NGF receptor	Neuroblastoma	[48,49]
FGF2	GM1	↓ Proliferation	CHO	[50]
PDGF	GM1	↓ Proliferation	Swiss 3T3	[32]
EGFR moves to caveolae	GM1	↓ Proliferation	Human breast epi- thelial	[51]
TrkA	GM1	↑ Neuronal differentiation	Neuro2A	[52]
↑ Akt, Erk1/2 phosphorylation	GD3 + HGF collagen 1	↑ Proliferation	Melanoma N1	[53]
Paxillin	GD3	↑ Migration	Melanoma N1	[7]
Mediates propagation of CD95-induced apoptosis	GD3	↑ Apoptosis	Lymphoblasts	[54]
Siglec-7 receptor on NK cells	GD3	↓ Immuno-suppressive	Natural killer	[55]
PDGFRα complexes with Yes kinase	GD3	↑ Proliferation and invasion	Glioma	[56]
Src	GD2	↑ Neurite retraction	Neuroblastoma	[57]
P13K/Akt mTOR	GD2	↑ Proliferation	Neuroblastoma	[58]
VEGF	GD1a	↑ Proliferation	HUVECs	[8]
HGF	GD1a	↓ Motility	FBJ osteosarcoma	[59]
Caspase-8,7 and PARP	GD1b	↓ Proliferation and ↑ apoptosis	Human breast cancer MCF-7	[60]
uPA	GT1b	↑ Apoptosis	Lung cancer A549	[61]
↓ interleukin 8 promoter	GQ1b	↓ Proliferation	Human melanoma	[62]

**^a^ Arrows** pointing up indicate an increase, those pointing down a decrease in growth factor response shown. **^b^ Growth factor** abbreviations: Akt, protein kinase B; VEGFR-2, vascular endothelial growth factor receptor 2; EGF, endothelial growth factor; FGF2 or bFGF, basic fibroblast growth factor; PDGFB, homo-dimer of the B subunit of platelet-derived growth factor; uPA, urokinase-type plasminogen activator; FAC, focal adhesion kinase; Src, proto-oncogene tyrosine-protein kinase; TGF-β1, transforming growth factor-β1; Trk, tyrosine receptor kinase (TrkA is activated by nerve growth factor, NGF); Erk1/2, extracellular regulated kinase; Yes, *homolog of the Yamaguchi sarcoma virus oncogene*; PI3K, phosphatidylinositol-3 kinase; mTOR, mammalian target of rapamycin; HGF, hepatocyte growth factor; and PARP, poly-ADP ribose polymerase. **^c^ Cell type** abbreviations: HUVEC, human umbilical vein endothelial cells; BHK, baby hamster kidney fibroblasts; CHO, Chinese hamster ovary; Neuro2A, mouse neuroblastoma2A; and FBJ, Finkel-Biskis-Jinkins murine osteosarcoma virus transformed nonproducer rat cells.

**Table 4:** Gangliosides recognized by siglecs.

Siglec	Cell Type Expressed on	Ganglioside Bound	Reference
1 (CD169)	Macrophage	GM3, GD1a, GD1b, and GT1b fairly equally	[118]
2 (CD22)	Primarily B cells	Strong preference for Neu5Nac- and Neu5Gcα2-6Gal	[119]
3 (CD33)	Mitogen-activated T and natural killer (NK) cells	GM3, GD3, GQ1b, GT1b (α2-3 and α2-6 sialylated gangliosides)	[120,121]
4 Myelin-associated glycoprotein	Myelinating cells	GD1a, GT1b, stabilizes axon-myelin interactions	[122]
5 (CD170)	T cells	GQ1b, weakly to GT1b	[123]
7 (CD328)	NK cells	GD3, GD2, GD1b, GT1b (preferentially binds α2-8 sialylated gangliosides)	[94,111,124]
8	Eosinophils and mast cells, less on basophils	Low affinity to GM2, GM3, GD3, GT1b, GQ1b	[123]
9 (CD329)	Monocytes, neutrophils, lesser amounts of NK, B, and T cells	GD1a, GT1b	[124]
10 (CD330)	Eosinophils, monocytes, subpopulation of NK cells	Only GT1b	[123,125]

## Data Availability

Not applicable.
